# Viridiflorol induces anti-neoplastic effects on breast, lung, and brain cancer cells through apoptosis

**DOI:** 10.1016/j.sjbs.2021.10.026

**Published:** 2021-10-13

**Authors:** Maaged A. Akiel, Ohoud Y. Alshehri, Shokran A. Aljihani, Amani Almuaysib, Ammar Bader, Ahmed I. Al‐Asmari, Hassan S. Alamri, Bahauddeen M. Alrfaei, Majed A. Halwani

**Affiliations:** a Department of Clinical Laboratory Sciences, College of Applied Medical Sciences, King Saud Bin Abdulaziz University for Health Sciences (KSAU-HS), King Abdullah International Medical Research Center (KAIMRC), Riyadh, Saudi Arabia; b Nanomedicine Department, King Abdullah International Medical Research Center/ King Abdulaziz Medical City, Ministry of National Guard, Health Affairs, Riyadh, Saudi Arabia; c Stem Cells and Regenerative Medicine, King Abdullah International Medical Research Center/ King Saud bin Abdulaziz University for Health Sciences, Riyadh, Saudi Arabia; d Faculty of Pharmacy, Umm Al-Qura University, Makkah 21955, Saudi Arabia; e King Abdul-Aziz Hospital, Laboratory Department, Ministry of Health, Jeddah, Saudi Arabia; f Department of Human and Molecular Genetics, Virginia Commonwealth University, Richmond, VA, United States

**Keywords:** Anticancer, Natural product, Apoptosis, Viridiflorol, Cytotoxicity, Drug discovery

## Abstract

All active natural molecules are not fully exploited as therapeutic agents, causing delays in the advancement of anticancer drug discovery. Viridiflorol is a natural volatile element that may work as anti-cancer compound. We tested the anticancer properties of viridiflorol at different concentrations ranging from 0.03 to 300 μM *in vitro* on three cancer cells including breast (MCF-7), lung (A549) and brain (Daoy). The cancer cells responses were documented after treatment using MTT and Annexin V assays. Viridiflorol showed cytotoxic effects against all tested cell lines, reducing cell viability in a concentration-dependent manner with variable IC_50_ values. Daoy and A549 cell lines were more sensitive to viridiflorol when compared with temozolomide and doxorubicin, respectively. Viridiflorol demonstrated the highest anticancer activity against the Daoy cells with an estimated IC_50_ of 0.1 µM followed by MCF-7 at 10 µM, and A549 at 30 µM. In addition, upon exposure to concentrations ranging from 30 µM to 300 µM of viridiflorol, early and late apoptotic cell death was induced in a concentration dependent manner in Daoy (55.8%-72.1%), MCF-7 (36.2%-72.7%) and A459 (35%-98.9%) cell lines, respectively. In conclusion, viridiflorol demonstrates cytotoxic and apoptotic ability in three different cancer cell lines (brain, breast and lung).

## 1 Introduction

Cancer is considered as multiple diseases causing a high mortality rate. The World Health Organization (WHO) estimated that 13% of the global annual mortality in 2018 were cancer-related (Sung et al., 2021). Consequently, the need for drug discovery has increased exponentially over recent decades (Takebe et al., 2018). The use of conventional chemotherapeutic agents in cancer therapy is no longer effective as several reports shown trends of resistance with significant toxicity (Al-malky et al., 2020). For example, doxorubicin is considerd the first line of treatment for breast cancer patients. Doxorubcin is a type of anthracyclines that blocks topo isomerase 2, a DNA repair enzyme. Results from patients has shown significant cardiotoxicity to breast cancer patients (Harris and Hochhauser, 1992). Similarly, temozolomide a genotoxic (DNA damaging) agent used as a first line of treatment for brain cancer patients have adverse effects on brain cancer patints (Choi et al., Sep. 2018); (Balana, Dec. 2020). The use of natural products as a source of novel cancer therapeutics has previously gained interest in the scientific community after the discovery of a range of promising candidates. One such group are terpenoids (Newman et al., 1981). Terpenoids include several classes of compounds comprising monoterpenes, sesquiterpenes, diterpenes, sesterterpenes, and triterpenes which showed a range of anticancer activities (Abdalla et al., 2020; Bader et al., 2019, D’Ambola et al., 2019, Dal Piaz et al., 2009 Laszczyk, 2009). Sesquiterpenes are attractive candidates for anticancer drug development due to their cytotoxic properties. Examples of sesquiterpenes include artemisinin, thapsigargin, and parthenolide, which are currently being tested in clinical trials (Ghantous et al., 2010). Viridiflorol is a natural volatile sesquiterpenes which was isolated for the first time in 1937 from Australian aromatic tree *Melaleuca viridiflora* Sol. ex Gaertn., it has cyclopropa[e]azulen skeleton (Hancox and T., and H. Jones, 1937). Previous studies indicated that viridiflorol has cytotoxic, anti-inflammatory, anti-oxidant, and anti-microbial activities (Trevizan, 2016). The essential oil of *Senecio rowleyanus* Jacob contains viridiflorol (11%), it has shown marked cytotoxic activity against brain cancer cell lines U251. Also it has antimicrobial activity against gram-positive and gram-negative microorganisms (El Hawary et al., 2008). Plants from Lamiaceae family are a good source of viridiflorol among them *Salvia algeriensis* Desf. (71.1%), *Satureja visianii* Šilić (17.9%) *Mentha aquatica L.* (11.3%), *Ballota undulata* (Sieber ex Fresen.) Benth*.* (6%) (Medjahed et al., 2016; Vidic et al., 2009, Esmaeili et al., 2006 Bader et al., 2003). *Salvia leriifolia* Benth essential oil contains 4.1% of viridiflorol, the oil was screened against different human cancer cell lines such as human caucasian lung large cell carcinoma COR-L23, human malignant melanoma A375, human renal cell adenocarcinoma ACHN, human amelanotic melanoma C32, human breast cancer cell line MCF-7, human prostate carcinoma LNCaP, in addition to human skin fibroblast 142BR as healthy human cells. The tested essential oil of *Salvia leriifolia* resulted in IC_50_ values of below 10 µg/ml against most of the screened cancer cells, which was regarded as more potent cytotoxicity compared to the activity of most of the isolated components alone. This was attributed by that sudy team to the possible synergy between the main and minor components of *Salvia leriifolia* (Loizzo, et al., 2010). According to literature, many essential oils containing variable percentages of viridiflorol were screened for their cytotoxic activity, but up-to-date no study was performed with viridiflorol alone as a potential anticancer agent. The objective of this study is to test anti-proliferative activity and apoptosis-inducing ability of viridiflorol in cancer cells originating from different organs.

## 2 Materials and methods

### 2.1 Chemicals and reagents

Viridiflorol, Doxrubicine, Temozolomide and dimethylsulphoxide (DMSO) were purchased from Sigma Chemical Co. (St. Louis, MO, USA) and dissolved in DMSO (final volume during cell manipulations were kept ≤ 0.1%) according to the manufacturer’s requirements. A real-time Glo MT Cell Viability Assay was purchased from Promega, Co. (Madison, WI, USA. Cat. G9711). Penicillin streptomycin, trypsin, phosphate buffer saline (PBS), DMEM media and fetal bovine serum (FBS) were purchased from Gibco (Grand Island, NY, USA).

### 2.2 Cell lines and culture condition

Breast cancer cell line (MCF-7, HTB-22), desmoplastic cerebellar medulloblastoma cell line (Daoy, HTB-186), and lung cancer cell line (A549, CCL-185) were ordered from ATCC. Each of the cell lines were maintained in Dulbecco modified Eagle medium–low glucose (DMEM) media containing 100 U/mL penicillin, 100 µg/mL streptomycin, and supplemented with 10% heat-inactivated fetal bovine serum (FBS). Cells were propagated in a humidified cell culture incubator with air 95% and CO_2_ 5% (v/v) at 37 °C. Experiments were done when expansion reaches 70% confuency. Culture conditions have been published previously (Alrfaei et al).

### 2.3 Assessment of cell viability

Cancer cell cytotoxicity of viridiflorol was assessed according to previously reported MTT method (Shaheen et al., 2018). Briefly, exponentially growing cells were collected using 0.25% Trypsin-EDTA and seeded in 96-well plates at 1x10^3^ cells/well. Cells were treated with viridiflorol at 0.03–300 µM for 24 h at 37 °C. Temozolomide was used at 0.16–5 mM for 24 h at 37 °C. Doxorubicin was used at 0.01–100 µM. Then the cells were exposed to the MTT for 1 h. Following the addition of DMSO to all wells containing variable degrees of purple formazan, which represent live cells, the absorbance was measured with multi-plate reader (BIORAD, PR 4100) A_555_, and was converted to percentage compared to 100% live cells in the vehicle control.

### 2.4 Assessment of apoptosis

Cells were seeded at 3x10^5^ for overnight in 6-well plates. Then, they were treated with viridiflorol at different concentrations according to the MTT result of each cell line for 24 h. Cell apoptosis was assessed using an Annexin V apoptosis kit according to previous reports (Alkahtani et al., 2019). Briefly, treated cells were harvested and washed twice with PBS and incubated in the dark with Annexin V-488/PI solution for 30 min at room temperature. After staining, cells were washed with PBS, after which the cells were subjected to a BD FACSCanto™ II flowcytometry (Becton, Dickinson Co., San Jose, CA, USA) and analyzed for 488 and PI fluorescent signals using the FL1 and FL2 signal detector, respectively (λex/em488/530 nm for 488, and λex/em 535/617 nm for PI). For each sample, 10,000 events were acquired and a positive 488 and/or PI cells were quantified by quadrant analysis and calculated using ACEA NovoExpress™ software (ACEA Biosciences Inc., San Diego, CA, USA).

### 2.5 Statistical analysis

Sigma plot for Windows, ver. 5.00 (software Inc., La Jolla, CA, USA) was used for data analysis, which were presented as mean ± SD. Analysis of variance (ANOVA) with a LSD post hoc test was devised for testing the significance using SPSS® for windows, version 17.0.0.p-value < 0.05 was considered as the cut-off value for significance.

Ethical Approval

The study was reviewed and approved by the Institutional Review Board (IRB) at King Abdullah International Medical Research Center (KAIMRC), Riyadh, Saudi Arabia with #Number #RC18/180/R.

## 3 Results

### 3.1 Viridiflorol anticancer activity toward breast cancer cell line

**Fig. 1:**
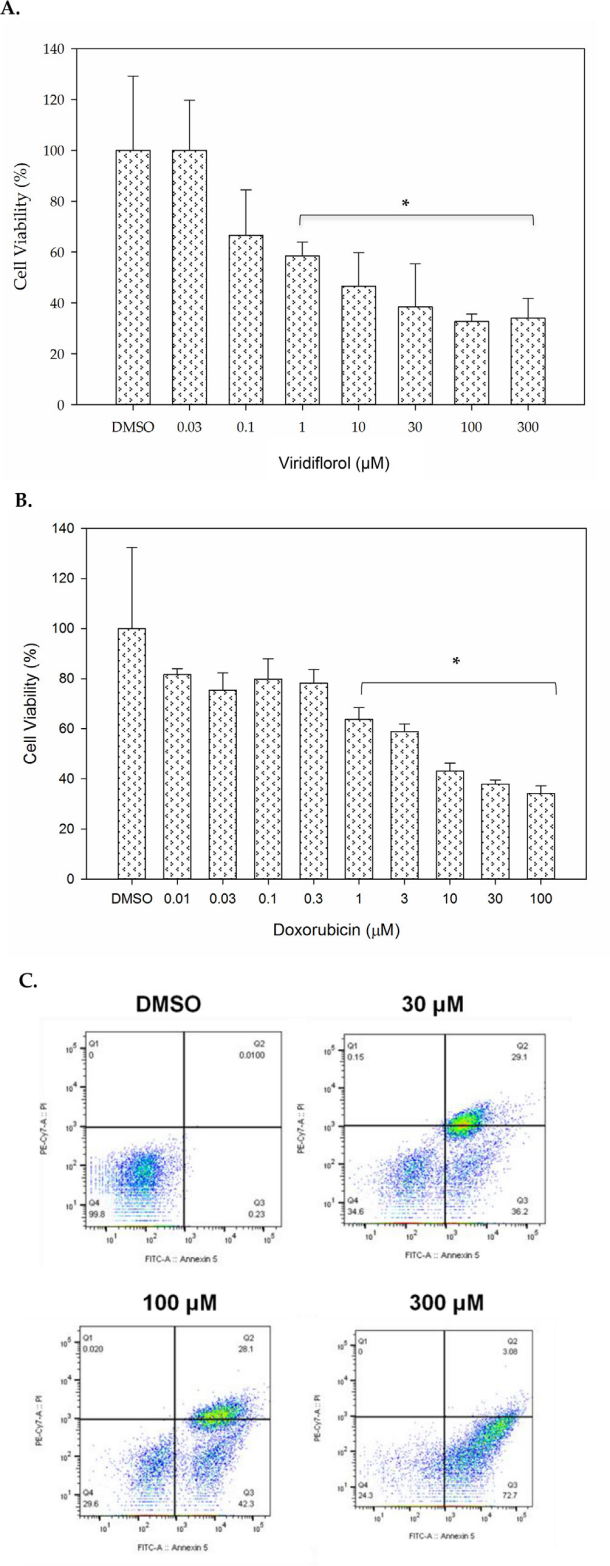
MTT assay showing cell viability of MCF-7 after treatment with virdiflorol (A) and Doxorubicin (B). DMSO was used as vehicle. Data represent ± SD (n = 3, * p-value < 0.05). The graph is a representative of three independent experiments. Flowcytometry analysis of MCF-7 after treatment with viridoflorol (C). X-axis: Annexin V and Y-axis: Propidium iodide. Data is representative of three independent experiments. Q1: necrosis (PI: positive, Annexin V: negative), Q2: late apoptosis (PI: positive, Annexin V: positive), Q3: early apoptosis (PI: negative, Annexin V: positive), Q4: live cells (PI:negative, Annexin V: negative).

MCF-7 cell line was used to investigate the anticancer activity of viridiflorol on breast cancer. Basically, MCF-7 cell line was treated with different concentrations of viridiflorol compound starting at 0.03 µM, 0.1 µM, 1 µM, 10 µM, 30 µM, 100 µM, and 300 µM. Cell viability post treatment (24 h) was measured by MTT assay (DMSO was used as vehicle). Viridiflorol was able to reduce cell viability and increase cytotoxicity in a concentration-dependent manner with an IC_50_ of about 10 µM compared to the vehicle-treated cells (Fig. 1A). Next, we used doxorubicin as a refernce drug to evaluate inhibition of cell viability on MCF-7. Treatment of doxorubicin significantly inhibited cell viability at concentrations ranging from 1 µM to 100 µM (Fig. 1B).

Furhtermore, to assess whether this reduction of viability by viridiflorol is due to the induction of apoptosis, we used Annexin V and Propidium iodide (PI) double staining. Thus, MCF-7 cells were treated with 30 µM, 100 µM and 300 µM of viridiflorol and DMSO as the vehicle control for 24 h and subjected to flowcytometry. We observed a noticeable increased percentage of early apoptosis which was represented by high Annexin V and low PI amounts cells in a concentration-dependent manner, showing 36.2%, 42.3% and 72.7% for the 30 µM, 50 µM, and 300 µM concentrations, respectively. The results confirmed that viridiflorol induced early apoptosis in the MCF-7 cell line (Fig. 1C). Thus, the reduction of MCF-7 cell viability happened through the induction of early apoptosis.

### 3.2 Viridiflorol anticancer activity on medulloblastoma cell line

**Fig. 2:**
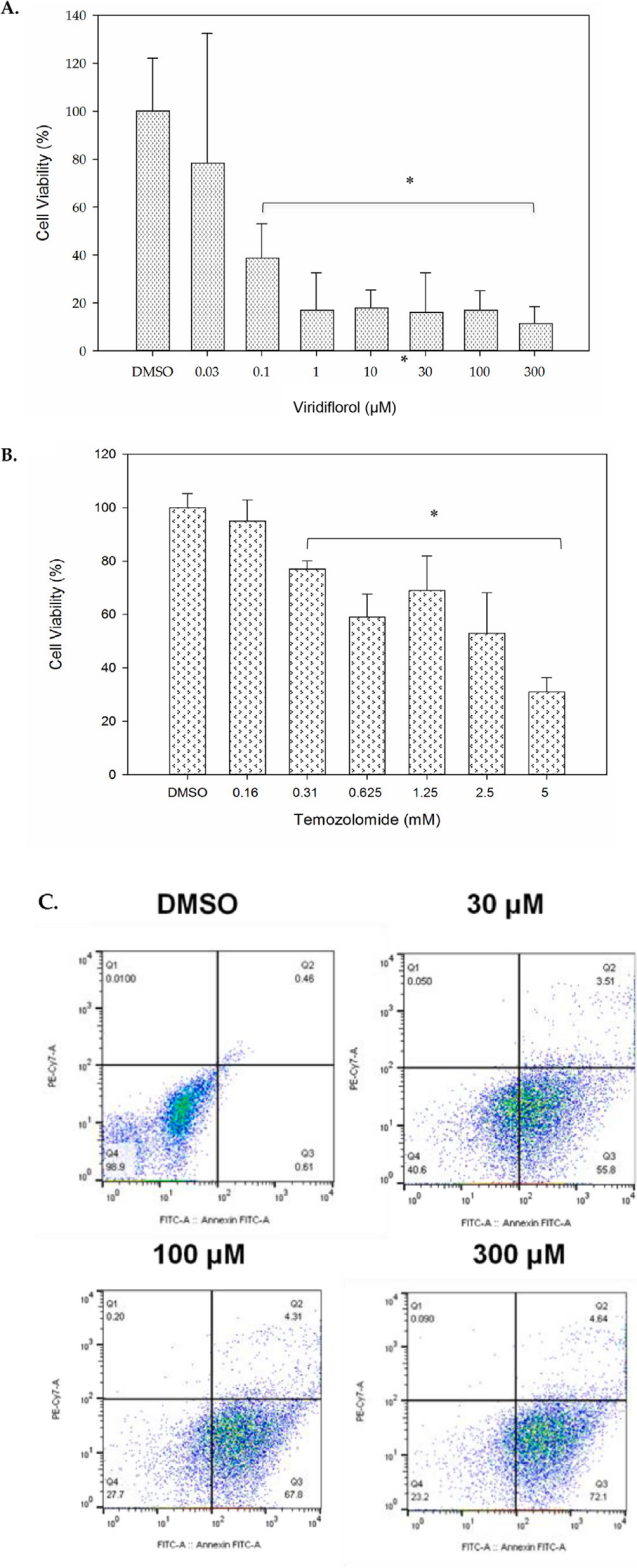
MTT assay showing cell viability of Daoy after treatment with virdiflorol (A) and Temozolomide (B). DMSO was used as vehicle. Data represent ± SD (n = 3, * p-value < 0.05). The graph is a representative of three independent experiments. Flowcytometry analysis of Daoy after treatment with viridoflorol (C). X-axis: Annexin V and Y-axis: Propidium iodide. Data is representative of three independent experiments. Q1: necrosis (PI: positive, Annexin V: negative), Q2: late apoptosis (PI: positive, Annexin V: positive), Q3: early apoptosis (PI: negative, Annexin V: positive), Q4: live cells (PI:negative, Annexin V: negative).

We have extended the evaluation of the cytotoxic effects of viridiflorol to the medulloblastoma cell line Daoy as a brain cancer cell line model. The medulloblastoma cell line was treated with different concentrations of the compound starting at 0.03 µM, 0.1 µM, 1 µM, 10 µM, 30 µM, and 100 µM, 300 µM and DMSO as the vehicle control for 24 h. The sensitivity of the Daoy cells to viridiflorol was higher than MCF-7 as it started to show significant inhibition of viability of more than 50% at 0.1 µM of viridiflorol (Fig. 2A). Thus, IC_50_ was estimated at 0.1 µM. Cell viability of Daoy cell line was then assessed using temozolomide, as a refernce drug for brain cancers. Temozolomide significantly inhibited viability at concentrations between 0.16 mM and 5 mM with highest inhibition observed at 5 mM concentration (Fig. 2B).

Next, we performed the Annexin V and PI double staining following the treatment of 30 µM, 100 µM, 300 µM of viridiflorol, and DMSO for 24 h to identify the cause of the inhibition of viability. Subsequently, the cells were subjected to flowcytometry. The results show that viridiflorol at 30 µM, 100 µM, and 300 µM induced early apoptosis (High Annexin V, low PI) at 55.8%, 67.8% and 72.1% (Fig. 2C). Collectively, the data show that the Daoy cell line is sensitive to viridiflorol induced apoptosis.

### 3.3 Viridiflorol anticancer activity on lung cancer cell line

**Fig. 3:**
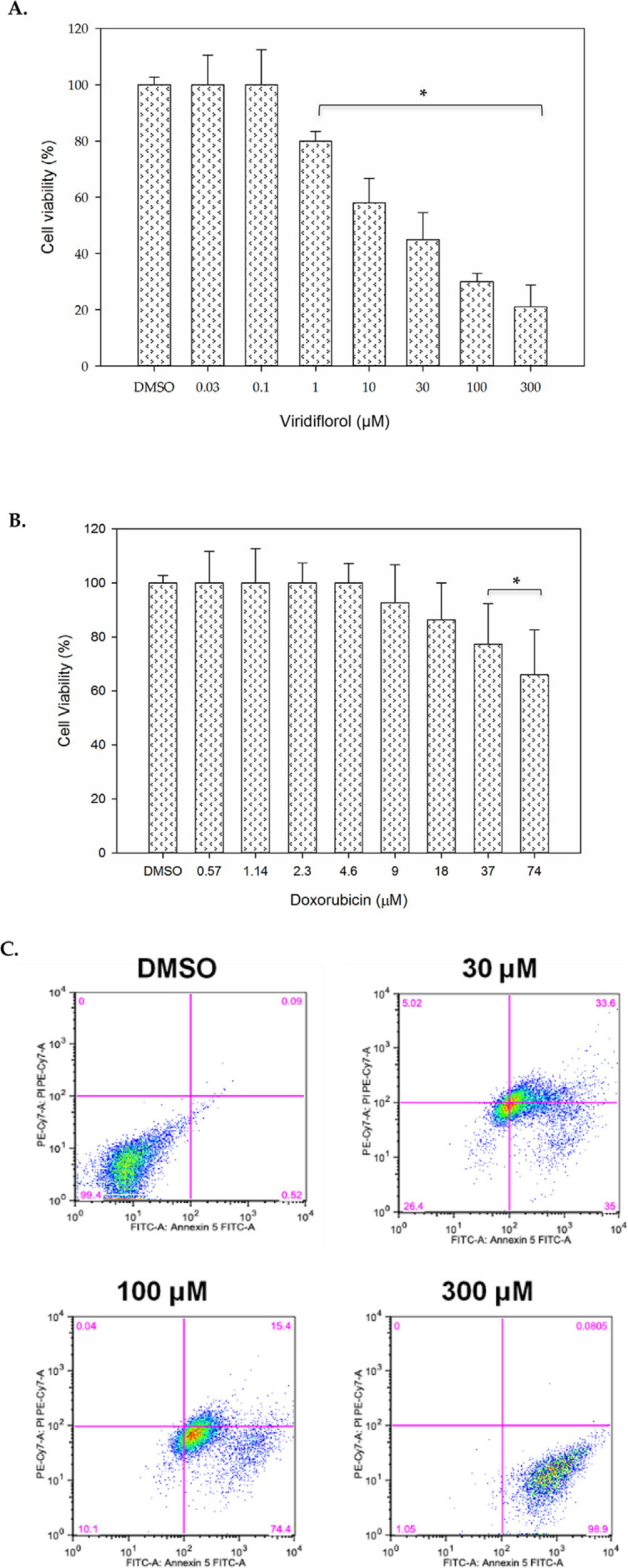
MTT assay showing cell viability of A549 after treatment with virdiflorol (A) and Doxorubicin (B). DMSO was used as vehicle. Data represent ± SD (n = 3, * p-value < 0.05). The graph is a representative of three independent experiments. Flowcytometry analysis of A549 after treatment with viridoflorol (C). X-axis: Annexin V and Y-axis: Propidium iodide. Data is representative of three independent experiments. Q1: necrosis (PI: positive, Annexin V: negative), Q2: late apoptosis (PI: positive, Annexin V: positive), Q3: early apoptosis (PI: negative, Annexin V: positive), Q4: live cells (PI:negative, Annexin V: negative).

Finally, we investigated the cytotoxic effects of viridiflorol on the lung cancer cell line A549, after treatment with different concentrations of the compound starting at 0.03 µM, 0.1 µM, 1 µM, 10 µM, 30 µM, and 100 µM, 300 µM and DMSO as the vehicle control for 24 h. Viridiflorol was able to significantly induce inhibition of cell viability in a concentration-dependent manner between 1 µM and 300 µM compared to vehicle treated cells with an estimated IC_50_ of 30 µM (Fig. 3A). Next, we were interested in comparing the cell viability of A549 to doxorubicin (Fig. 3B). Doxorubicin was able to significantly inhibit viability of A549 cells at 37 µM and 74 µM concentrations (Fig. 3B).

To validate the inhibition of viability, double staining with Annexin V and PI was used after treatment with 30 µM, 100 µM and 300 µM of viridiflorol and DMSO for 24 h after which cells were subjected to flowcytometery and analysis. The results indicated that viridiflorol remarkably induced high early apoptosis (high Annexin V, low PI) in a concentration-dependent manner at 35% (30 µM), 74.4% (100 µM), and 98.9% (300 µM) (Fig. 3C). Thus, provided evidence viridiflorol was able to induce apoptosis in A549 cells.

## 4 Discussion

Viridiflorol belongs to the class of aromadendrane sesquiterpenoids isolated from many aromatic plants (Ghantous et al., 2010)– (Bader et al., 2003). It was synthesized chemically in 1992 and tested against the fungi *Cladosporium cucumerinum* revealing fungitoxic activity (Gijsen et al., 1992). Since the plant extracts containing viridiflorol has shown previously a cytotoxic effects against cancer cell lines (El Hawary et al., 2008); (Loizzo, et al., 2010), it was our purpose in this study to test the cytotoxic effects of pure viridiflorol on multiple cancer cell lines.

Using natural products for the development and the treatments for a wide range of diseases, including cancer, has been successful for decades (Newman et al., 1981); (Cordell, 2000). In this study, we examined the *in vitro* cytotoxic effects of viridiflorol using cell culture on three cancer cell lines and determined the IC_50_ of viridiflorol on breast, medulloblastoma, and lung cancer cell lines. We provide evidence that viridiflorol has cytotoxic effects and is capable of inducing apoptosis in three cancer cell lines of different origins. The medulloblastoma Daoy cell line was the most sensitive to viridiflorol, followed by the breast cancer cell line MCF-7, and finally lung cancer cell line A549. The IC_50_ for the MCF-7 was estimated as 10 µM, for Daoy 0.1 µM, and for A549 30 µM. The observed inhibition of cell viability was compared with reference chemotheraptic agents. For breast and lung cancer cell lines we used doxorubicin as a reference drug. In the case of MCF-7 cell line, the inhibitory effect by doxorubicin at 1 µM to 100 µM was comparable to viridiflorol (Fig. 1A and B). Lower concentrations of doxorubicin between 0.01 µM and 0.3 µM were more potent than viridiflorol, however, it did not reach statistical significance (Fig. 1A and B). For the A549 cell line our results show that the sensitivity of A549 to viridiflorol was higher than doxorubicin as viridiflorol was able to significantly inhibit viability of A549 cell line at a concentration as low as 1 µM concentration compared to 37 µM and 74 µM of doxorubicin (Fig. 3A and B). The cytotoxic effects of viridiflorol on medulloblastoma cell line (Daoy) when compared with temozolomide show that the latter induced more than 40% inhibition at concentrations ranging between 0.625 mM and 5 mM. This is similar to the previously reported inhinbitory concentrations of temozolomide against daoy where the IC_50_ was determined to be 669.63 µM (Pezuk et al., 2015). The sensitivty of Daoy cell line to veridiflorol was higher than that of temozolomide as inhibition of viability by viridiflorol was significantly induced at micomolar concentrations as low as 0.1 µM (Fig. 2A and B).

The ability of anticancer agents to induce apoptosis is unpredictable due to variations in the molecular mechanisms regulating carcinogenesis in different cell type i.e. genetic heterogeneity (Fisher et al., 2013). Viridiflorol is a lipophilic molecule with topological polar surface area (TPSA) = 20.23 (Schepetkin et al), this parameter has been shown to correlate very well with blood–brain barrier penetration (Pajouhesh and Lenz, 2005). The blood brain barrier (BBB) is known to protect tumor components from therapy success. It imposes significant challenges to the brain tumors treatment**.** (Karmur, 2020)**.** Some studies reported that the essential oils rich in viridiflorol exerts anti-cancer effects and induce apoptosis, the essential oil of *Cyperus longus* contains viridiflorol as one of the major constituents (4.7%), the IC_50_ in MCF7 cells was 31.35% after 72 h, and 39.91% for PC3 cells, while the apoptosis was 78.23% and 65.35% for MCF7 and PC3 cells respectively (Memariani, 2016). The aromatic plant *Blepharocalyx salicifolius* contains several sesquiterpenes among viridiflorol (8.83%), the essential oil has exerted cytotoxic activity against the MDA-MB-231 (46.60 µg·mL − 1) breast cancer cell line at 46.60 µg·mL − 1 concentration, the oil does not induce cell death but it causes impairment of cellular metabolism of cancer cells (Furtado et al).

Apoptosis is irreversible programmed cell death, a homeostatic mechanism that cells can use to commit suicide to regulate and maintain proliferative homeostasis to keep cellular compartments uncrowded (Elmore, 2007). It is initiated by several mechanisms when cells are exposed to stimuli such as irreparable damage or toxic compounds (Elmore, 2007); (Norbury and Hickson, 2001). Evasion of apoptosis is regarded as a key hallmark of cancer (Hanahan and Weinberg, 2011).

When cells decide to undergo apoptosis the organization of phospholipids bilayer on plasma membrane are altered leading to the flipping of phosphatidylserine (PS) from the inner surface of the phospholipid bilayer to the outer surface. As a result, the surface exposed PS can be detected through its affinity for binding to Annexin V, a phospholipid binding protein, that can be assessed using flowcytometry (Coxon et al., 2011).

Double staining of cells using Annexing V and Propedium Iodide can be used to differentiate the percentage apoptotic to necrotic cell death that occurred after exposure to cytotoxic stimuli (Coxon et al., 2011). We confirmed the occurrence of apoptotic cell death following exposure of breast, medulloblastoma and lung cancer cell lines to viridiflorol. Our results show that viridiflorol triggered apoptosis by more than 70% after 24 h in all cell lines examined.

## 5 Conclusions

The use of conventional chemotherapeutic agents such as doxorubicin and temozolomide to cancer therapy provided modest increase in survival of patients diagnosed with breast, brain and lung cancers with significant toxicity to normal tissues (Pugazhendhi et al., May 2018); (Braun and Ahluwalia, 2017). Our data open the door for new route of anticancer therapy. Viridiflorol ability to inhibit multiple cancers bring an therapeutic option to toward patient outcome. In previous report the use of essential oils as an active components provided reduced toxicity to hamster fibroblsts when compared with cancer cell lines (Oliveira et al., 2015). These findings on viridiflorol are encouraging for future pharmacologic manipulation of the molecule to enhance its potency for oncological therapeutic approaches and to pave the path for the development of anticancer therapeutic agents with reduced toxicity towards normal cells (Oliveira et al., 2015). One limitation of this study is that cytotoxic effects of viridiflorol on normal cells will be evaluated in the future. Another limitation of our study is that we did not assess the therapeutic effects of viridiflorol *in vivo* using animal models of cancer. Moreover, the mechanisms causing the apoptotic events due to viridiflorol exposure are unkown. Those variables will be determined in future studies.

## Funding

This research was funded by King Abdullah International Medical Research Center (KAIMRC), grant number RC18/180R.

## CRediT authorship contribution statement

**Maaged A. Akiel:** Conceptualization, Investigation, Writing – original draft, Methodology, Writing – review & editing. **Ohoud Y. Alshehri:** Investigation, Writing – original draft. **Shokran A. Aljihani:** . **Amani Almuaysib:** Project administration, Investigation. **Ammar Bader:** Methodology. **Ahmed I. Al‐Asmari:** Validation. **Hassan S. Alamri:** Methodology, Validation, Visualization. **Bahauddeen M. Alrfaei:** Conceptualization, Writing – review & editing. **Majed A. Halwani:** Conceptualization, Project administration, Funding acquisition, Writing – review & editing, Supervision.

## Declaration of Competing Interest

The authors declare that they have no known competing financial interests or personal relationships that could have appeared to influence the work reported in this paper.
